# Anxiety and depression in patients with intracranial meningioma: a mixed methods analysis

**DOI:** 10.1186/s40359-022-00797-6

**Published:** 2022-04-08

**Authors:** Graham Kasper, Shannon Hart, Nardin Samuel, Colleen Fox, Sunit Das

**Affiliations:** 1 Faculty of Medicine, University of Toronto, Toronto, ON Canada; 2 Faculty of Medicine, Memorial University of Newfoundland, St. John’s, NL Canada; 3 Institute of Medical Science, University of Toronto, Toronto, ON Canada; 4 Division of Neurosurgery, St. Michael’s Hospital, University of Toronto, Toronto, ON Canada; 5 Person-Centred Care, Ontario Health (Cancer Care Ontario), Toronto, ON Canada

**Keywords:** Anxiety, Depression, Mixed-methods analysis, Meningioma, Watchful waiting

## Abstract

**Background:**

While diagnosis with a high-grade intracranial tumor is known to be associated with increased psychosocial burden, the burdens associated with meningioma are less well described. This study aimed to investigate the mental health burden in patients with meningiomas who have undergone surgical resection or serial observation, so as to identify and enhance awareness of gaps in care.

**Methods:**

The Hospital Anxiety and Depression Scale (HADS) was administered to participants. Fisher’s Exact tests were performed to evaluate frequency distributions and t-tests were applied to compare postoperative and non-surgical patients’ HADS scores. Semi-structured interviews were completed on a subset of participants. Thematic analysis of interviews identified emerging themes.

**Results:**

Thirty patients with intracranial meningiomas met inclusion criteria. The cohort’s mean age was 56.01 years and 66.67% were women (n = 20). Fourteen underwent surgery; sixteen were treated conservatively with observation. The average time since diagnosis of the sample was 37.6 months. Prevalence of mild to severe symptoms of anxiety was 28.6% amongst surgical management patients and 50% for active surveillance patients (*p* = 0.325). The prevalence of mild to severe symptoms of depression was 7.14% amongst surgical management patients and 6.25% for active surveillance patients (*p* = 0.533). Emerging themes from eight interviews reveal the influence of resilience, uncertainty and time, social support, interactions with medical experts, and difficulties during recovery on mental health.

**Conclusion:**

The findings from the present study reveal that patients with meningiomas experience a significant mental health burden, illustrating the need for enhanced patient-centred care focusing on mental health.

**Supplementary Information:**

The online version contains supplementary material available at 10.1186/s40359-022-00797-6.

## Background

Meningiomas are the most common primary intracranial tumor in North America, accounting for approximately a quarter of all non-metastatic lesions [1]. In comparison to other intracranial tumours, meningiomas often progress slowly, and generally hold a good prognosis: the 5-year survival rate and 15-year survival rate are as high as 85–90% and 75–80%, respectively [2–4]. Their slow rate of growth, combined with the increasingly widespread use of cranial imaging, leads to many patients being diagnosed incidentally and oftentimes without any associated symptoms. Surgery remains the mainstay of treatment for meningiomas and is often curative if gross total resection is achieved [4, 5]. However, many asymptomatic meningiomas are left untreated, and are instead monitored regularly through serial imaging [6]. Despite their favourable prognosis, there is a risk of recurrence after resection, particularly in higher-grade tumours [4].

Meningiomas are relatively understudied compared to their malignant counterparts [2]. This claim rings true when examining the mental and emotional well-being of patients with meningiomas compared to patients with higher grade brain tumours. For example, depression and anxiety are common in brain tumor patients, with the prevalence of depression ranging from 10 to 40%, and the prevalence of anxiety ranging from 5 to 50% [6–16]; however, the majority of studies investigating this relationship have focused on high-grade tumor populations, without significantly considering meningiomas [12–15].

Additionally, in comparison to patients with more aggressive tumours, patients with meningiomas have lower recurrence rates, often avoid adjuvant therapies, and have an extended period of long-term follow-up [17]. Each of these elements contribute to a unique patient experience. Given their relative preponderance compared to other brain tumours—cruelly compounded by their favourable prognosis and patients’ higher survival rates—the toll of mental health issues, such as anxiety and depression, on patients living with meningiomas is likely far greater than currently appreciated. Moreover, as the Canadian healthcare system continues to place a greater emphasis on providing holistic, patient-centred care, acknowledging the mental and emotional well-being or distress that patients with meningiomas may face must be part of the new standard of care [18]. Thus, it is necessary to evaluate and understand the burden of symptoms of anxiety and depression in this patient population to ultimately improve the quality of care they receive.

Furthermore, research that has explored the burden of depression and anxiety on patients with meningiomas has relied primarily on quantitative data [5, 19]. The lack of qualitative analysis, exploring patients’ perspectives concerning the effect of their diagnosis, treatment and follow-up care, has likely contributed to the dearth of conclusive results in this field of study. Qualitative methods add depth and context to the breadth of information quantitative data provides and they are prominently employed in mental health research [20]. Advantages of using such an approach in this patient population include enhancing the scientific community’s understanding of the unique experiences patients with benign brain tumours go through, directly eliciting patients’ personal perspectives, and informing new hypothesis generation for future research.

Combining quantitative and qualitative methods provides an improved understanding of the collected data than either approach alone [21]. This serves as an excellent tool to study symptoms of anxiety and depression in patients with low-grade meningioma as there is a need to explore the depths and context of these symptoms (best captured by qualitative analysis), while also obtaining complementary and converging data outlining the prevalence and intensity of these symptoms (best captured by quantitative analysis).

To these ends, we performed a mixed-methods study using quantitative and qualitative analysis to identify the burden and prevalence of symptoms of depression and anxiety in patients with meningioma. We sought to compare these symptoms and patient experiences between those who were treated with surgery and those who underwent serial observation.

## Materials and methods

### Study design

An explanatory sequential mixed-methods design was employed. Patients identified during recruitment were informed of the study during a Neurosurgery Clinic visit, or by telephone. After obtaining informed consent, participants completed the HADS questionnaire immediately, or scheduled a future telephone assessment. The results were de-identified.

A subset of twelve participants, five who had surgery and seven who pursued watchful waiting (WW), were randomly identified to partake in the interview phase. Interviews, with only the interviewer and interviewee present, were performed and recorded via telephone. Recordings were then transcribed, de-identified, and analyzed. Four individuals identified to participate in the interview phase of the study did not complete interviews (three for logistical reasons and one who withdrew consent for this phase of the study). The interview process continued until a satisfactory level of thematic saturation was reached, which occurred following eight interviews (surgery = 5, WW = 3) [22]. All assessments and interviews were administered and documented by the first author.

Participants were partitioned into a Surgery or Watchful Waiting subgroup for the purpose of comparison.

### Participant selection

The study was approved by the Unity Health Toronto Research Ethics Board, and all research was performed in accordance with relevant guidelines, including the Declaration of Helsinki. Adult patients with intracranial World Health Organization (WHO) Grade I or II meningiomas under care of the primary investigator (SD) were considered possible participants. This decision was made secondary to logistical constraints. From July 2018 to February 2019, 56 individuals were contacted and 32 agreed to participate. For those participants who were recruited during remote/telephone appointments, verbal consent was obtained, otherwise written consent was obtained from all other participants. This procedure was approved by the Ethics Board. Exclusion criteria were: diagnosis of anxiety and/or depression pre-existing a diagnosis of meningioma, non-fluency in English, and cognitive impairment preventing comprehension and communication of the study and its aims. Two patients were withdrawn for having a prior diagnosis of anxiety and/or depression.

### Outcome measures

#### Anxiety and depression

The Hospital Anxiety and Depression Scale (HADS) was used to detect symptoms of anxiety and depression [23–26]. The HADS consists of 14 questions designed to evaluate anxiety and depression (7 questions each). Each question is scored from 0 to 3, with 3 representing worse symptomatology, generating one score for anxiety and another for depression. Scores ≤ 7 indicate a normal score, scores ≥ 11 indicate clinically relevant anxiety or depression, and scores from 8 to 10 indicate mild anxiety or depression. The HADS demonstrated high internal consistency reliability with a Cronbach’s α of 0.816 for anxiety and 0.776 for depression.

Semi-structured interviews were conducted to investigate participants’ perspectives concerning their mental health (Additional File 1: Interview Guide). This format allowed for flexibility to explore relevant topics that emerged freely from discussion. Questions focused on participant’s mental health and well-being, specifically concerning symptoms of anxiety and depression, as well as their general concerns, access to or lack of support networks, and satisfaction or dissatisfaction with their cumulative patient experience.

### Additional measures

#### Sociodemographic and clinical data

Age, gender, sex, tumor grade and location, presenting symptoms (neurological deficits), treatments employed (surgery, radiation, serial monitoring), and treatment outcomes (extent of resection) were recorded from the medical record.

### Statistical analysis

Statistical analyses were conducted using SPSS Statistics version 27 (IBM Corporation, Armonk, NY). Summary statistics were calculated for the cohort and each subgroup. A Fisher’s Exact Test for categorical variables was conducted to assess for statistical significance of frequency distributions of normal scores (≤ 7) vs. mild/high scores (> 7). Independent sample *t*-tests assuming unequal variances were conducted to compare mean anxiety and depression HADS scores between the subgroups. Statistical tests were considered significant if the *P* value was less than 0.05 (two-tailed).

### Thematic analysis

Qualitative data was evaluated using a descriptive thematic data analysis approach. Open coding was generated inductively, in parallel, and independently by two investigators for each interview transcript, labelling ideas, concepts and themes in the text [27]. Emerging themes were refined, employing iterative comparison analyses to identify patterns and develop relationships between concepts established in the transcripts [27]. Using axial coding, data across transcripts were linked to reveal final themes and sub-themes [27]. We then compared their analyses and generated overarching themes and sub-themes describing the sum of the data [27]. The analyses were then re-reviewed by our co-investigator experienced in qualitative research.

## Results

Thirty participants were included in the study, with 14 (46.7%) in the Surgery subgroup and 16 (53.3%) in the WW subgroup. There were no significant differences in demographics between the two groups. Table 1 outlines all the summary statistics in detail.

**Table 1:** Participant demographic and medical information

Variable	Post-surgical resection, n (%)	Watchful waiting, n (%)
**Count**	14 (46.67)	16 (53.33)
**Age (years)**		
Range	39–80	33–84
Mx (Sx)	55.71 (10.59)	56.31 (14.35)
**Gender**		
Men	5 (35.71)	5 (31.25)
Women	9 (64.29)	11 (68.75)
**Tumour grade**		
WHO Grade I	11 (78.57)	–
WHO Grade II	3 (21.43)	–
**Extent of tumour resection**		
Total	12 (85.71)	–
Subtotal	2 (14.29)	–
**Tumour lateralization**		
Right	6 (42.86)	11 (68.75)
Left	5 (35.71)	3 (18.75)
Other (bilateral, unspecified)	3 (21.43)	2 (12.50)
**Tumour location**		
Cerebellopontine Angle	0 (0)	2 (12.50)
Convexity	3 (21.43)	2 (12.50)
Falcine	3 (21.43)	3 (18.75)
Foramen Magnum	1 (7.14)	0 (0)
Olfactory Groove	1 (7.14)	2 (12.50)
Parasagittal	0 (0)	1 (6.25)
Posterior Fossa	1 (7.14)	2 (12.50)
Sphenoidal	3 (21.43)	2 (12.50)
Tentorial	1 (7.14)	2 (12.50)
Other (bilateral, multiple)	1 (7.14)	2 (12.50)
**History of radiation therapy**		
Yes	3 (21.43)	2 (12.50)
No	11 (78.57)	14 (87.50)
**Presenting symptoms**		
Headache	8 (57.14)	7 (43.75)
Motor Deficit	8 (57.14)	3 (18.75)
Seizure	3 (21.43)	1 (6.25)
Vertigo/Dizziness/Unsteadiness	4 (28.57)	3 (18.75)
Visual deficit	5 (35.71)	4 (25.00)
**History of Neurological Risk Factor (HTN, DLD, Smoking)**		
Yes	6 (42.86)	6 (37.50)
No	8 (57.14)	10 (62.50)

*Mx* statistical mean, *Sx* standard deviation, *HTN* Hypertension, *DLD* Dyslipidemia

### Frequency and intensity of anxiety and depression

The prevalence of mild to clinically relevant anxiety (HADS scores > 7) amongst the study cohort was 40%; within the Surgery subgroup the prevalence was 28.6%, and within the WW subgroup it was 50%. The prevalence of mild to clinically relevant depression (HADS scores > 7) amongst the cohort was 6.67%; within the Surgery subgroup, the prevalence was 7.14%, and within the WW subgroup it was 6.25%. Frequency distributions of anxiety and depression positive-cases (scores > 7) and non-cases (scores ≤ 7) did not differ significantly between the Surgery and WW subgroups (anxiety: *p* = 0.325, depression: *p* = 0.533) (Table 2). There was no significant difference in mean anxiety or depression scores when comparing the two subgroups (anxiety: *p* = 0.587, depression: *p* = 0.798) (Table 2).

**Table 2:** Results regarding anxiety and depression

HADS	Post-surgical Resection		Watchful waiting		Total	
	n (%)	Raw scores, *M (SD)*	n (%)	Raw scores, *M (SD)*	n (%)	Raw scores, *M (SD)*
HADS A	–	6.21 (3.91)	–	7.06 (4.54)	–	7 (4.00)
Low (≤ 7)	10 (71.43)	4.2 (2.20)	8 (50)	3.25 (2.66)	18 (60)	3.78 (2.39)
Moderate/High (> 7)	4 (28.6)	11.25 (2.06)	8 (50)	10.88 (1.96)	12 (40)	11 (1.91)
HADS D	–	3.21 (2.99)	–	2.94 (2.85)	–	3.07 (2.88)
Low (≤ 7)	13 (92.86)	2.77 (2.59)	15 (93.75)	2.4 (1.96)	28 (93.33)	2.57 (2.23)
Moderate/High (> 7)	1 (7.14)	9	1 (6.25)	11	2 (6.67)	10 (1.41)

*M* statistical mean, *SD* standard deviation, *HADS* Hospital Anxiety and Depression Scale

### Relationship of time on anxiety and depression

At the time of assessment with the HADS questionnaire, the range in time since diagnosis for the entire cohort was 2 to 182 months, with a mean time since diagnosis of 37.6 months. Linear regression analysis was performed to evaluate the impact of time since diagnosis on HADS scores. While scores trended downward with increased time since diagnosis, no significant relationship was revealed (*p* = 0.217 for Depression and *p* = 0.113 for Anxiety).

### Thematic analysis

Mean interview length was 41.9 min. Thematic analysis revealed four common themes: personal resilience, the impact of uncertainty and time, the importance of social support, and mental health difficulties during recovery. The interview participants’ summary statistics are outlined in Table 3.

**Table 3:** Interview participants’ select demographic information and HADS scores

Variable	Post-surgical resection, n (%)	Watchful waiting, n (%)	Total
**Count**	5 (62.5)	3 (37.5)	8
**Age (years)**			
Range	46–68	33–52	33–68
Mean	52.8	43	49.1
**Gender**			
Men	2 (40)	0 (0)	2 (25)
Women	3 (60)	3 (100)	6 (75)
**Presenting symptoms**			
Headache	2 (40)	3 (100)	5 (62.5)
Motor Deficit	3 (60)	2 (66.7)	6 (75)
Seizure	2 (40)	0 (0)	2 (25)
Vertigo/Dizziness/Unsteadiness	0 (0)	1 (33.3)	1 (12.5)
Visual Deficit	2 (40)	1 (33.3)	3 (37.5)
**Time since diagnosis (months)**			
Range	15–66	6–64	6–66
Mean	42.6	27.3	36.9
**Time since surgery (months)**			
Range	15–65	–	–
Mean	42.4	–	–
**HADS A > 7**	2 (40)	3 (100)	5 (62.5)
**HADS D > 7**	1 (20)	1 (33.3)	2 (25)

*HADS* Hospital Anxiety and Depression Scale

#### Theme 1: Personal resilience

This theme encapsulates participants’ ability to cope with their diagnosis and rebound during their recovery period. Six interviewees specifically mentioned having a self-proclaimed positive outlook on life: “Being positive is the one thing I would have to say is important” (Participant B1). Participant A4 highlighted an example of this optimism when they said, “emotionally, even though there’s lots of things to deal with… you can dwell on that, or you can dwell on the fact that, ‘Hey, I’m going to have supper tonight.’” Optimism was noted to be a longstanding trait amongst participants—“my default setting is to be happy and positive and… look on the bright side of things” (Participant A5)—and was frequently attributed to family values and upbringing: “we make the best out of whatever situation [we’re] in, and that’s… always carried [our family]” (Participant A4).

#### Theme 2: The impact of uncertainty and time

A substantial component of the diagnostic and follow-up periods involved dealing with a degree of uncertainty, which was a catalyst for anxiety in participants. Participants reported a heightened sense of worry and fear associated with unknowns and lack of information. Delays in assessment following initial diagnosis were a noted cause of distress: “it was probably more stressful for that [reason], because of the period where you have to wait to find out what the result is… It’s stressful to worry about, ‘Oh my god, what’s going on with me?’” (Participant B1). Participant B14 described the fear associated with unknowns: “[I] started to worry… because I don’t know why it’s there, or how it’s happened, or anything like that, so it was very frightening.” A similar scenario occurs during follow-up for both surgically and conservatively managed patients: “[A lack of information] definitely alters… my moods and how I function leading up to [the appointment]” (Participant B14). The impact of uncertainty or lack of knowledge is emphasized by reported alleviation of stress once more information becomes available: “the more information I had about it, the better I felt” (Participant B14).

#### Theme 3: Importance of social support

This theme captures the significant benefit patients’ felt from having meaningful social support, the detrimental effects that isolation and loneliness had on mental well-being, and the desire for support from peers who have gone through similar experiences. There was a strong association between social support and resilience: “There’s more people out there… who care about you… and override… the situation that you’re in” (Participant A4). Having close friends willing to listen was an important source of emotional support: “Talking with friends and… getting off my chest what was going on… It was often a release to tell people… and for them to… be there for me and listen and sympathize” (Participant B17).

A prevalent theme was the desire for additional resources in the way of peer support, in order to connect with people who have gone through something similar. Seven of eight interviewees stated that they would have found a peer support group helpful, specifically early on in the diagnosis.

#### Theme 4: Recovery difficulties

Following the initial diagnosis and management, interviewees discussed navigating a period of time lasting weeks to months which was particularly stressful and difficult. These struggles were related to lack of coordinated care, residual deficits, and the desire to return to premorbid levels of functioning.

Participant concerns regarding lack of coordinated care centred on communication issues between healthcare professionals and patients. Participant B1, for example, experienced lingering visual symptoms after surgery and reported concern that they had not heard from their ophthalmologist or primary care physician after initial visits for assessment. Several participants felt that connections to outpatient rehabilitation services were never adequately established, and that they had to navigate these resources independently.

In relation to struggling with residual deficits, participants discussed their desire to return to baseline functioning: “[My focus is] getting back to… being normal” (Participant A9). The struggle with being unable to return to prior levels of functioning was distressing: “when someone takes away that normal… you got to fight to get back to normal” (Participant A5). Despite accepting the idea of a new “normal,” Participant A3 described difficulty in adjusting to their new state, for example, dealing with memory issues and acclimating to new thresholds of tolerable social stimulation.

## Discussion

To our best knowledge, this study is the first to investigate symptoms of anxiety and depression in patients with low-grade meningiomas (WHO Grade I or II) using a mixed-methods design. Our study captured quantitative data to assess the prevalence of mild and clinically relevant symptoms of depression and anxiety and complemented this with rich qualitative data from semi-structured interviews, allowing for comprehensive analysis of patient mental health. Our findings demonstrate that patients with low-grade meningiomas experience many of the same challenges as individuals with high-grade tumors, especially in the short term; thus, they may share similar needs and suffer from similar psychologic and psychiatric morbidity.

### Prevalence and intensity of anxiety and depression

The prevalence of anxiety amongst the cohort was 40% (n = 12). It is difficult to compare point prevalence with lifetime or annual prevalence, metrics often cited in literature; however, our findings suggest a greater prevalence of depression and/or anxiety than compared to the general Canadian population, whose prevalence of generalized anxiety and/or major depression ranges from 9.4% to 11.3% [28]. Further broken down into anxiety and depression, we observed a prevalence of 40% (n = 12) for anxiety and 6.67% (n = 2) for depression.

#### Analysis of anxiety in patient cohort

We found that 50% (n = 8) and 28.6% (n = 4) of participants within the WW and Surgery subgroups, respectively, demonstrated moderate/high HADS anxiety scores (> 7). These values are well above the annual and lifetime prevalence of generalized anxiety in Canada, which, according to the Statistics Canada Canadian Community Health Survey on Mental Health (CCHS-MH), are 2.6% and 8.7% respectively [29]. This finding confirms what others have shown; despite their benign nature, meningiomas are associated with significant psychiatric comorbidity, specifically symptoms of anxiety [5, 19]. The prevalence of anxiety amongst our cohort is also comparable to levels described in malignant tumor patient cohorts [5, 8, 11, 30, 31]. However, studies that compared the difference in prevalence of anxiety over time between malignant and benign brain tumor patients have found that, amongst meningioma patients, rates of anxiety decrease and normalize within 5 years [19, 32]. Overall, our findings are in accordance with the literature, and describe rates of anxiety similar to those suffered by malignant brain tumor patients around the period of initial diagnosis [30, 33].

Interestingly, there was no significant difference in the mean HADS anxiety scores between the Surgery and WW subgroups. This finding could suggest three important points: (1) in patients with slow-growing tumors, curative treatment may not be the driving factor influencing improvements in mental and emotional well-being; (2) the benefits of curative surgery on anxiety may be counteracted by the detrimental effects of post-operative recovery, healing, and residual deficits; and (3) the watchful waiting approach may be associated with significant stress and psychiatric co-morbidity over time, despite only being implemented for cases with favourable prognoses.

#### Analysis of depression in patient cohort

The prevalence of depression in the Surgery and WW subgroups were 7.14% (n = 1) and 6.25% (n = 1), respectively. These scores indicate a potentially higher burden of depression in patients with low-grade meningiomas than in the general Canadian population, where the annual prevalence of depressive episodes has been determined to be approximately 5% [29, 34]. Our findings are in line with previous data published by Goebel and Mehdorn [19], who described a prevalence of depression of 8% in their meningioma patient study cohort.

While there is consensus that rates of depression are higher in patients with intracranial tumors than the general population—ranging as high as 38%—data comparing differences between tumor grade is less uniform [9, 10, 30, 34]. Pringle et al. [11] found that patients with meningiomas had higher depression scores when compared to patients with high-grade tumors; conversely, several studies have shown the opposite [8, 9, 11]. These inconsistencies may reflect the effect of time on patients’ mental health throughout their treatment course. There may be a peak in the rate of depressive symptoms in patients with meningiomas around the time of diagnosis, pre-operatively and perioperatively, as reported by Bommakanti et al. [31] and Simoca et al. [35]. This peak in symptoms was followed by an improvement in depression scores post-operatively [36–38]. This contrasts with findings discussed by D’Angelo et al. [7] and Litofsky et al. [33], who demonstrated an increase in prevalence of depression over time in high-grade tumor patients [7, 33]. Our findings are congruent with other studies demonstrating stable depression levels over time in meningioma patients [19]. Moreover, our analysis did not reveal any significant difference in the prevalence of depression or mean depression scores when comparing the Surgery and WW subgroups. Our findings suggest that in patients with meningioma, symptoms of depression are caused not only by the mere presence of the tumor, and that struggles during recovery and the management of lingering symptoms following surgery may mask any benefit of cure on rates of depression.

### Emergent themes from patient interviews

#### Positive impacts on patient well-being

In our study, factors that tended to contribute to a positive patient experience and mental well-being included personal resilience and strong social support. This is unsurprising, as resilience has been noted to play an important role in the restoration of physical and emotional well-being in cancer patients and has been thought to be associated with the adoption of coping strategies that improve quality of life [39–41]. Social support is a major contributor to resilience, and there is substantial evidence that it is essential for maintaining both physical and psychological health [42]. Interviewed participants expressed immense gratitude for the emotional support of friends and family. Nonetheless, it was indicated that there was occasionally a lack of understanding or empathy from these support figures, suggesting a role for peer support [43]. Participants indicated that talking to others with analogous experiences would be helpful [17]. Prior studies have found that the sense of camaraderie and kinship that develops from peer support intervention increases hope, decreases loneliness, and encourages a positive change in perspective and values [44–46].

#### Negative impacts on patient well-being

Uncertainty, lack of support from the healthcare system, and managing difficulties during recovery contributed negatively to patient well-being. Uncertainty has been shown to be detrimental to health-related quality of life in patients with high-grade brain tumors; our study supports the conclusion that uncertainty is also a major contributor to stress and anxiety in patients with benign tumors [47, 48]. All patients also reported a period of adjustment to physical and cognitive deficits. Acceptance of the “new normal” was associated with a better self-rated mental health. The acknowledgement of overcoming a life-defining event such as a meningioma diagnosis significantly improves self-perception and decreases anxiety; however, prolonged deficits may hamper this self-perception of strength and resilience [49].

#### Impact of time on well-being

Time was an overarching factor that played a dynamic role in the patient experience. Most pertinently, the amount of time that had passed since diagnosis was an important factor in the patients’ well-being. While the quantitative results did not reveal a significant difference in HADS scores as a function of time since diagnosis, interviewees reported having an initial period during recovery that was both physically and emotionally challenging. Previous reports have shown that objective measures of anxiety decrease over time in meningioma patients who have undergone resection, and this was reflected in our interviews [19, 49].

#### Implications for clinical practice and health systems planning

Several suggestions for improving future clinical practice and health systems planning can be inferred from the results of this study. Firstly, peer support should be offered to patients early on in diagnosis. Linking individuals to established programs would prove beneficial at the patient level and may be instituted practically at a systems level, rather than establishing small-scale supports unique to each hospital. Participants indicated that this support would be most useful early on after diagnosis, but it would likely also be helpful in promoting optimism and overcoming difficulties during recovery. Additionally, individualized care and subsequent tailoring of resources should be emphasized by physicians. The creation and dissemination of health literate and plain language resources should also be a goal. Going forward, well established metrics already used by many cancer treatment centers, such as the Hospital Anxiety and Depression Scale, could be implemented into care plans. As rehabilitation services are paramount to recovery, access to these resources—particularly in the outpatient setting—should be secured for all patients who require them. Finally, despite the lack of prolonged clinical anxiety or depression, we found that patients often experienced a period of emotional difficulty for several months after resection, and patients should be made aware of this possibility.

### Limitations

Our study was a retrospective analysis with a small sample size, which impedes the generalizability of findings and may influence assessment of the study’s outcome measures. Furthermore, the study was completed at a single center with patients under the care of a single surgeon. The patient experience may differ at other centers and thus impact the accuracy of our results. In addition, HADS scores were collected at a single time-point during the patients’ follow-up, rather than at several points, precluding the opportunity to study changes in mental health status over time. Moreover, only one subset of patients with brain tumors was analyzed with no comparison to patients with higher-grade tumors, or to healthy controls. Recruiting both high-grade brain tumor and healthy control groups would highlight meningioma specific conclusions. Finally, larger cohorts are essential to decipher the impact of presenting symptoms, such as headache and visual deficits, on mental health.

## Conclusion

Taken together, this study found that a significant portion of patients with intracranial low-grade meningiomas have symptoms suggestive of anxiety and/or depression. Undergoing surgery versus pursuing serial monitoring did not impact the prevalence or intensity of anxiety or depression within our patient cohort. Factors influencing mental well-being and mental distress in our patient cohort included personal resilience, the element of uncertainty, particularly while waiting for follow-up assessments, difficulty adjusting to residual symptoms or deficits, and the overarching effect of time since diagnosis or post operation. Future studies with larger cohorts should assess anxiety and depression longitudinally at multiple timepoints, using multiple metrics to evaluate symptom severity. This will enable further characterization of the psychiatric burden this patient population faces and will lead to a better understanding of relationship of time on their psychiatric symptoms.

## Supplementary Information

**Additional file 1**. Interview Guide.

## Data Availability

The datasets generated and/or analysed during the current study may be made available from the corresponding author on reasonable request.

## Abbreviations

CCHS-MH: Canadian Community Health Survey on Mental Health
HADS: Hospital Anxiety and Depression Scale
WHO: World Health Organization
WW: Watchful Waiting
