# Supplementary material for: Anxiety and depression in patients with intracranial meningioma: a mixed methods analysis
Source: BMC Psychol. 2022 Apr 8;10:93. doi: 10.1186/s40359-022-00797-6 (PMC8994241; doi:10.1186/s40359-022-00797-6)
Supplement: Supplementary file 1 — Additional file 1. Interview Guide. [file 40359_2022_797_MOESM1_ESM.docx]

**Appendix:** Anxiety and depression in patients with intracranial meningioma: a mixed methods analysis – interview guide.

**Baseline Information**

1. How old are you?
2. How would you identify your gender?
3. When were you diagnosed with a meningioma?
4. Have you had curative surgery to remove the meningioma?
   1. If yes, how old were you when you had surgery to remove the meningioma?
5. How did you discover that you had a meningioma?
6. What was your initial reaction to this discovery? If you feel comfortable doing so,

please take me through that process.

**Patient Experience, Mental Well-Being, Depression and Anxiety**

1. What do you think of your own mental health?
2. Have you ever been diagnosed with a mental health disorder or illness? If yes, and you feel comfortable doing so, please elaborate.
3. Are you receiving support or specific treatment for your mental health? If yes, and you feel comfortable doing so, please elaborate.
4. Have you noticed changes in your mental health throughout the course of treatment, from diagnosis to follow-up appointments?
   1. If you experience symptoms of depression or anxiety, what is the trigger for their onset?
5. How has your diagnosis affected your day-to-day life?
6. How did you feel about your prognosis for the future? (i.e., did you feel anxious or sad about the future?)
7. Did you feel like you had adequate support from the healthcare team (e.g., family physician, surgeon, oncologist, neurologist, allied health professionals etc.) treating you?
8. Did you feel like you had an adequate social support network in place?
9. How did you feel the physicians treating you perceived your diagnosis and prognosis?
   1. Did their perspective of the disease and its impact on your life align with your own perspectives? (i.e., Were their concerns in line with yours/were they understanding of, and in tune with, your concerns?)
10. What were your concerns before surgery? If you have not had surgery, what are your concerns with the watchful follow-up approach?
    1. Were those concerns addressed to your satisfaction? Please explain.
11. Please describe the follow-up care you received after surgery. If you have not had surgery, describe the on-going care you have received during follow-up.
    1. After surgery, how did your patient experience change? (i.e., in terms of quality/frequency of care etc.). If you have not had surgery, how has your patient experience changed during the follow-up period?
12. After surgery, how would you describe your levels of stress and anxiety? If you have not had surgery, how would you describe your levels of stress and anxiety throughout the follow-up period?
13. Are you concerned or worried with the risk of recurrence/ongoing growth? If yes, please describe this concern/worry. If yes, has the healthcare team currently providing you care inquired about your concerns of recurrence?
14. After surgery, how would you describe your motivation and interest or pleasure in doing things? If you had not had surgery, how would you describe your motivation and interest or pleasure in doing things throughout the follow-up period.
15. After surgery, how would you describe your emotional well-being? If you have not had surgery, how would you describe your emotional well-being throughout the follow-up period.
16. How has your surgery affected your life and outlook for the future? If you have not had surgery, how has your continued follow-up affected your life and outlook for the future?

**Final Comments**

1. Do you have any additional comments you would like to add?
